# The impact of the human microbiome in tumorigenesis, cancer progression, and biotherapeutic development

**DOI:** 10.1186/s12866-022-02465-6

**Published:** 2022-02-12

**Authors:** Claire M. Doocey, Karen Finn, Craig Murphy, Caitriona M. Guinane

**Affiliations:** 1 Department of Biological Sciences, Munster Technological University, Cork, T12 P928 Ireland; 2 Department of Analytical, Biopharmaceutical and Medical Sciences, School of Science and Computing, Galway-Mayo Institute of Technology, Dublin Road, Galway, H91 T8NW Ireland

**Keywords:** Human microbiome, Cancer, Genetic loci/mechanisms, Cytotoxic, Tumour microenvironment, Microbial therapies, Probiotics

## Abstract

**Background:**

Cancer impacts millions of lives globally each year, with approximately 10 million cancer-related deaths recorded worldwide in 2020. Mounting research has recognised the human microbiome as a key area of interest in the pathophysiology of various human diseases including cancer tumorigenesis, progression and in disease outcome. It is suggested that approximately 20% of human cancers may be linked to microbes. Certain residents of the human microbiome have been identified as potentially playing a role, including: *Helicobacter pylori, Fusobacterium nucleatum, Escherichia coli, Bacteroides fragilis* and *Porphyromonas gingivalis.*

**Main body:**

In this review, we explore the current evidence that indicate a link between the human microbiome and cancer. Microbiome compositional changes have been well documented in cancer patients. Furthermore, pathogenic microbes harbouring specific virulence factors have been implicated in driving the carcinogenic activity of various malignancies including colorectal, gastric and pancreatic cancer. The associated genetic mechanisms with possible roles in cancer will be outlined. It will be indicated which microbes have a potential direct link with cancer cell proliferation, tumorigenesis and disease progression. Recent studies have also linked certain microbial cytotoxins and probiotic strains to cancer cell death, suggesting their potential to target the tumour microenvironment given that cancer cells are integral to its composition. Studies pertaining to such cytotoxic activity have suggested the benefit of microbial therapies in oncological treatment regimes. It is also apparent that bacterial pathogenic protein products encoded for by certain loci may have potential as oncogenic therapeutic targets given their possible role in tumorigenesis.

**Conclusion:**

Research investigating the impact of the human microbiome in cancer has recently gathered pace. Vast amounts of evidence indicate the human microbiome as a potential player in tumorigenesis and progression. Promise in the development of cancer biomarkers and in targeted oncological therapies has also been demonstrated, although more studies are needed. Despite extensive *in vitro* and *in vivo* research, clinical studies involving large cohorts of human patients are lacking. The current literature suggests that further intensive research is necessary to validate both the role of the human microbiome in cancer, and the use of microbiome modification in cancer therapy.

## Introduction

Cancer imposes a significant burden on global health and life expectancy. A comprehensive review of GLOBOCON’s cancer incidence and mortality statistics based on 185 countries and 36 cancers, estimated 19.3 million cancer cases and approximately 10 million cancer related deaths worldwide in 2020 [1]. Concerningly, it was also documented that the international cancer impact is expected to reach 28.4 million cases by 2040. Notably, female breast cancer (BC) (2.3 million new cases) has surpassed lung cancer as the main cause of malignancy worldwide, with gastrointestinal cancers such as colon, gastric, liver and pancreatic all listed in the top 15 malignancies affecting populations in 2020 [1].

Risk factors associated with cancer development include: familial genetics, lifestyle factors, obesity, smoking, nutrient deficiencies, alcohol consumption, UV exposure and infectious agents [2]. However, in 2018 an estimated 2.2 million infection-related cancers were diagnosed worldwide [3], and it has been suggested that approximately 20% of human cancers are a potential consequence of infectious pathogens [4–6]. Oncogenic microorganisms have been identified and extensively researched over the past few decades, with 11 infectious pathogens listed as grade 1 carcinogens by the International Agency for Research on Cancer (IARC) [3]. Four of these agents, *Helicobacter pylori*, high-risk human papilloma virus (HPV), hepatitis B virus and hepatitis C virus account for 90% of infectious cancer cases globally [3]. Notably, *H. pylori*, identified in 1982, is currently the only bacterial species on this list that is implicated in gastric disease, and was the first bacterium to be associated with cancer [7]. However, a number of bacterial residents of the human microbiome are now emerging as potential cancer-causing microbes. Notable examples under current investigation include: *Fusobacterium nucleatum* in colorectal cancer (CRC) [8–10], *Porphyromonas gingivalis* in pancreatic cancer (PC) [11] and oral cancer [12], both associated with the oral microbiome, and gut microbiome residents *Escherichia coli* and *Bacteroides fragilis* in CRC [5, 13, 14] respectively.

The human microbiome is an intrinsic element in the lifelong maintenance of health and of immune system homeostasis [15, 16]. Mounting research has now recognised the human microbiome as a key area of interest in relation to the advancement of tumorigenesis, cancer progression and in disease outcomes [10, 17–20]. Recent studies have also suggested the role of bacterial virulent gene toxins and metabolites, that may severely dysregulate essential cellular processes or facilitate the ability of proteins to invade and adhere to various organ tissue leading to the onset and progression of cancer [17, 21–23]. Given the cytotoxic nature of certain bacterial gene products, specific species have also shown the potential capacity for use in cancer treatment regimens [24–27].

Taken together, this review aims to provide a comprehensive analysis of the emerging role of our resident microbiota in human oncogenesis, with a focus on key bacterial species that have gained recent scientific attention. We will evaluate the molecular mechanisms that have been proposed for these agents to understand the genetic loci involved in cancer development and progression. Furthermore, the possibility to use our microbiota as potential biotherapeutic agents will also be discussed.

## The human microbiome in cancer

The human microbiome contains approximately 100 trillion microbial cell residents [28]. The vast majority of these microbes are located in the gut, with *Bacteroides, Firmicutes, Actinobacteria* and *Proteobacteria* phyla representing the four major bacterial populations in the human gastrointestinal microbiota [28, 29]. The adult microbiome composition is influenced by host genetics, diet, environment and factors including antibiotic use which can alter gut balance [30]. Any shift in the microbial composition can have an adverse effect on the human host, and may influence the onset of various conditions including cancer [30].

Recent research indicates that certain residents of the oral, lung, gastric, duodenal, pancreatic and colorectal human microbiome niches, play a role in oncogenesis and tumour advancement. There is vast evidence indicating specific bacterial taxa and their associated genetic loci in cancer development and progression which is discussed in detail in Section Genetic determinants implicated in Cancer development. As mentioned, previous studies have established a causal link between gastric resident *H. pylori* and gastric cancer (GC) [7]. The gut bacterium *B. fragilis* was linked to CRC in a 2009 study [13], while *E. coli* was associated with CRC following analysis by researchers in 2012 [31]. The oral pathogen *F. nucleatum* was also identified as a potential causative agent in CRC by two unrelated teams of scientists in 2012 [8, 9], and research has been mounting implicating *F. nucleatum* in CRC in more recent years (Section Fusobacterium nucleatum).

Additionally, many studies have suggested association between variance in human bacterial composition and cancer. Compositional variance in the gut microbiome of CRC patients when compared to the gut microbiome of benign individuals and variances in the tumour microbiome of CRC tissue and non-cancer tissue has been documented [32, 33], respectively. Ahn and colleagues discovered decreased bacterial diversity in the faecal samples of CRC subjects. These samples were found to be depleted of *Firmicutes* with lower abundance of the class *Clostridium* and possessed higher levels of the pro-inflammatory genera *Fusobacterium* and *Porphyromonas* [32]*.* Hibberd et al. conducted a study with fifteen CRC patients and twenty-one healthy controls. An enhanced microbial diversity and an increase of numerous taxa in the intestinal tumour microbiota including *Fusobacterium, Selenomonas* and *Peptostreptococcus* was noted when compared with the intestinal mucosal biopsies from the non-cancer cohort. *Fusobacterium* was the most prevalent genus discovered in the tumour microbiota, the mean abundance in tumour tissue was > 7% and < 0.5% in control sample tissue [33].

It has been established that many tumours harbour bacteria, suggesting that tumours are non-sterile [33–35]. Microbial alterations in the pancreas, an organ historically perceived to be sterile, has been observed. It was understood that bacterial species were unable to tolerate the highly alkaline environment or the proteases found in the pancreatic juices [36]. However, recent discoveries of large microbial communities within the pancreatic tissue of PDAC (Pancreatic Ductal Adenocarcinoma) patients when compared to the pancreatic tissue of healthy individuals, may play a role in pancreatic carcinogenesis [34]. This data showed that the phyla *Proteobacteria, Bacteroides* and *Firmicutes* were the most abundant in all human PDAC tissue specimens with *Actinobacteria* also associated with each sample. The genera *Pseudomonas* and *Elizabethkingia* were prevalent in all the human PDAC tissue samples evaluated [34].

Furthermore, in a recent extensive study, tissue from over 1,500 samples from 7 different human tumour types were examined and compared, and it was deduced that variations between the different tumour types were evident [35]. These included 1,010 tumour samples and 516 normal tissue samples, mainly from adjacent cell tissue. The 7 human tumour samples studied were breast, lung, melanoma, ovary, brain, bone and pancreatic. Each tumour type investigated had a unique tumour microbiome. Additionally, 22 CRC samples were added to the study for comparison purposes [35]. *Proteobacteria* and *firmicutes* phyla accounted for most of the detected bacterial sequences across all cancer types examined [35]. The *Proteobacteria* to *Firmicutes* ratio varied between the tumour samples. Breast tumour samples were found to have 3 main phyla: *Proteobacteria, Firmicutes* and *Actinobacteria. Actinobacteria* phylum, namely, *Corynebacteriaceae* and *Micrococcaceae* family members were detected in the non-gastrointestinal tumours. Bacteria from the *Firmicutes* and *Bacteroides* phyla were most abundant in CRC samples whilst *Proteobacteria* was dominant in the PC microbiome. *F. nucleatum* was common not only in CRC tissue as anticipated, but also in pancreatic and breast tumour tissue [35]. Nejman et al. also confirmed the presence of bacteria within the cytoplasm of both tumour cells and immune cells during this study, validating the idea that the tumour environment is non-sterile [35].

Compositional variance within the well-established female genital tract microbiome has also been investigated [37]. Discrepancies in composition of this localised microbiota has been linked with various pathologies, namely, cervical cancer, sexually transmitted disease and bacterial vaginosis. *Porphyromonas somerae* and *Atopobium vaginae,* when present together, are a potential cause for concern in relation to endometrial cancer progression [37]. An *in vitro* study found that these bacteria have the potential to promote expression of cytokines (including IL-1α, IL-1β, IL-17α and TNFα) and chemokines (including CCL13, CLL8 and CXCL2), thus inducing an inflammatory status that could encourage the onset of cancer in the endometrial environment [37]. However, limitations of this study were acknowledged, in that an endometrial cell line was utilised when infecting with the bacterial species, rather than primary healthy tissue from the endometrium. This suggests that further investigation is needed to confirm this concept. Notably, recent studies also support the suggestion that the microbiome composition of BC tissue may vary from healthy breast tissue [38–40]. However, evidence as to whether an individuals’ microbial constitution contributes to, or potentially causes BC remains inconclusive.

Studies have suggested that the healthy human lung compromises four main phlya of bacterial species, *Actinobacteria, Firmicutes, Bacteroides* and *Proteobacteria* with *Prevotella* and *Veillonella* also prevalent [41–43]. It has been suggested that the balance of this local microbiome may become compromised in the presence of disease [41–43]. Lung cancer has been linked to imbalances in the lung microbiota and has been the focus of both human studies [44] and animal studies [45]. Lee et al. assessed the bronchoalveolar lavage fluid (BAL) microbiome of 28 human participants (20 with lung cancer and 8 benign individuals) and found a significant increase in two phlya in lung cancer patients, *Firmicutes* and *TM7*. They also discovered that the genera *Veillonella* and *Megasphera* were significantly more abundant in the cancer cohort. These researchers subsequently suggested these genera as potential biomarkers for lung cancer [44]. Jin et al. used a human lung adenocarcinoma murine model to demonstrate that commensal lung bacteria have a role to play in lung tumorigenesis. Disease development was linked to an increase in total bacterial burden and an associated decreased bacterial diversity in the bronchial airways of lung tumour bearing mice when compared to healthy tumour free mice. Notably, *Herbaspirilium* and *Sphingomonadaceae* were found to be more abundant in the lungs of mice with tumour growth [45].

Although compositional changes within specific habitats have been associated with carcinogenesis, there is a possibility that these may be relational rather than causative [46]. Nevertheless, there is mounting evidence of certain taxa such as *F. nucleatum*, *P. gingivalis*, *E. coli* and *B. fragilis,* supporting their contribution to the onset and development of various cancers, as highlighted in recent reviews [47–51]. We will review the evidence of genetic determinants associated with these microbes and their associated link to human malignancies.

## Genetic determinants implicated in cancer development

Certain bacterial species of the human microbiome have the ability to secrete pathogenic products. This was highlighted in a recent review which suggested that these potent secretions are implicated in cell apoptosis, genotoxicity, immune defense evasion, inflammatory processes and ultimately cancer evolution [52]**.** Pathogenic bacteria can interact with the human host triggering chronic inflammation and subsequent damage that in turn has been associated with tumorigenesis. Inflammatory processes and the stimulation of inflammatory signaling pathways have been linked with pathogen-related inflammation in cancer [53].

Specific virulence factors have been identified as the potential gene loci important in driving the carcinogenic activity of various malignancies. These include those produced by bacterial species including *H. pylori, F. nucleatum* and *B. fragilis,* and *P. gingivalis*, implicated in GC, CRC and PC respectively.

### Gastric cancer

*Helicobacter Pylori* was designated a WHO Group 1 carcinogen in 1994, one of 11 infectious organisms with this notable distinction [3]. Approximately, 90% of GC’s are caused by *H. pylori* with 50% of the global population colonised [54]. However, carriers may remain asymptomatic and avoid development of malevolent disease. It is estimated that 1% of *H. pylori* infection results in GC [55]*.*

*H. pylori* is an established causal factor in gastric malignancy and its carcinogenic activity is driven by several virulence factors. The most studied to date include *cagA* (Cytotoxic- associated gene A), *vacA* (Vacuolating cytotoxin A) and OipA (Outer inflammatory Protein) [23], (See Table 1). *H. pylori* is divided into type I and type II strains differing due to the presence of a 40 kb pathogenicity island containing the CagA protein and VacA toxin in type I strains [56]. These differences influence the pathogenicity of this bacterium and its ability to potentiate disease [57]. Evidence suggests that type I strains are associated with an increased risk of GC development and other gastric pathologies while type II strains are not [56, 58].

**Table 1:** Summary of key bacterial genetic loci with potential links to the onset and development of cancer based on recent literature

Bacterial Species	Gene product	Mechanism of action	Potential associated cancer	References
*Helicobacter pylori*	CagA	Dysregulation of Wnt/β catenin signalling cascade. Activation of NF-kB, leads to gastric inflammation and dysplasia	Gastric cancer	[59, 60]
*Helicobacter pylori*	VacA and OipA	Induce gastric epithelial apoptosis which is associated with ulceration and aberrant cell proliferation in gastric tissue, leading to metaplasia and oncogenesis	Gastric cancer	[61]
*Fusobacterium nucleatum*	FadA	Adheres to E-cadherin (a tumour suppressor) and stimulates Wnt/β catenin pathway. Inflammatory process leads to proliferation of colorectal cancer cells	Colorectal cancer	[62]
*Escherichia coli*	Colibactin	DNase activity, creates double stranded breaks within epithelial cells. Promotes cell proliferation and tumour maturation	Colorectal cancer	[5, 18]
*Escherichia coli*	Colibactin A (*clbA*)	*clbA* encodes a phosphopantetenyl transferase, subsequently modifying the three polypeptide synthases of the *Pks* locus	Colorectal cancer	[63]
	Colibactin P *(clbP*)	*clbP* codes for peptidase and splices a precursor of colibactin (pre-colibactin) into its active form		
*Bacteroides fragilis*	Bft	Stimulates colonic stat 3 activity and Th17 mucosal immune response. Cleaves E-cadherin, triggers β-catenin nuclear signalling. Linked to colon cell hyperplasia and cell proliferation	Colorectal cancer	[13, 64]
*Porphyromonas gingivalis*	PPAD	Can instigate arginine degradation, which antagonises *P53* gene and *KRAS* mutation	Pancreatic cancer	[65]
*Porphyromonas gingivalis*	Gingipains	Promotes activation of MMP-9 proenzyme into its mature form leading to tumour cell proliferation	Oral cavity metastasis	[66]

Recent studies have reemphasized the relationship between *cagA, vacA* and *oipA* with gastritis and increased GC risk [20, 67]. The mechanistic role of CagA in carcinogenesis has been investigated using various animal model systems including zebrafish [55], transgenic mice [68] and gerbils [59]. These studies all suggest that CagA positivity induces the development of gastric adenocarcinoma. Specifically, CagA functions through injection into gastric epithelial cells by a type IV secretion factor (T4SS) and once *in-situ* impacts upon intracellular transduction systems enabling the reorganisation of the actin cytoskeleton [69]. CagA also instigates the release of the pro-inflammatory cytokine IL-8 [60] which leads to subsequent alterations in the gastric epithelium and the possible initiation of inappropriate cell proliferation [55, 59]. This sustained inflammation can lead to chronic gastritis and tumorigenesis [55, 59]. VacA, also an important toxin, can facilitate colonisation of *H. pylori* in gastric cells and is affiliated with disease progression [70]. VacA is suggested to increase urease activity via its ability to create cellular vacuoles, allowing diffusion of urea from mucosal tissue to the gastric lumen. This action enables the survival of *H. pylori* in the acidic gastric environment and prevents cellular apoptosis [71]. Both CagA and VacA are well established virulence factors in relation to *H. pylori*; however, the synergistic action between the two remains uncertain [72, 73]. The OipA protein encoded by the *oipA* gene may also participate in IL-8 stimulation and chronic inflammation [74]. A member of a large family of outer membrane proteins, it may work synergistically with CagA in the development of GC [61].

Whilst *H. pylori* has been identified as a causal agent in GC its relationship with CRC requires further investigation. Epplein et al. found that five *H. pylori* proteins, particularly VacA protein increases the risk of CRC by 60–80%, but found that the presence of *H. pylori* alone does not increase risk [75]. However, the results of a prospective multicentre European study released in 2020 identified a link between *H. pylori* seropositivity (51% of CRC cases), the *vacA* gene (36% of CRC cases), the Helicobacter cysteine rich protein (28% of CRC cases), and an elevated chance of acquiring CRC [76].

### Colorectal cancer

CRC represents the third most diagnosed malignancy and the second most likely cause of oncological related death globally [77]. Mounting research has highlighted the role of the microbiome in generating intestinal inflammation and inducing colonic tumorigenesis [21, 22]. Widely acknowledged pathogenic species of bacteria implicated in this pathology include *F. nucleatum* [8, 9], *E. coli* [31] and *B. fragilis* [13]. The role of these three principal microbial pathogens was explored in a recent review [50]. All three microorganisms are residents within the human microbiome.

#### Fusobacterium nucleatum

*Fusobacterium nucleatum* is a member of the oral microbiome and a traditional periodontal pathogen. It has been correlated historically to periodontitis, appendicitis, and more recently to CRC [8, 9]. It is known to migrate to distant sites when linked to aggressive pathology, including CRC [78]. Enrichment of *F. nucleatum* has been observed in CRC tissue in numerous studies when compared to healthy adjacent colonic tissue [78, 79]. It has been identified as a potential novel therapeutic target and prognostic biomarker for this malignancy, particularly when associated with advanced disease stages [78].

Cell surface proteins of *F. nucleatum,* FadA (Fusobacterium adhesion A) (Table 1), Fap2 (Fibroblast activation protein 2) and *radD* (Radiation gene), are virulence factors, implicated in CRC [80]. The FadA protein which is necessary for the adhesion and subsequent invasion of *F. nucleatum* into host cells was characterised in 2005 [81]. Dispersion of FadA adhesion complex beyond the oral mucosa is associated with inflammation and cell proliferation [62]. Rubenstein and colleagues investigated the genetic mechanisms of FadA that drives CRC, as per Table 1 [62].

Fap2 and RadD, both autotransporter protein family members, also play an important role in the virulence and adhesive nature of *F. nucleatum* [82]*.* Colon-targeted tumour recognition by *Fusobacteria* is associated with Fap2. It identifies and attaches to tumours displaying Gal-GalNAc (acetylgalactosamine), which is overexpressed in both colorectal adenocarcinoma and CRC metastasis [10]. Fap2 has a protective mechanism through the inactivation of natural killer cells against malignant cells, caused by the suppression of the TIGIT receptor [10]. RadD is associated with cell death and cell–cell aggregation [83].

Investigative research by Tomkovich et al. 2017, found that FadA and Fap2 adhesion of *F. nucleatum* in APC (Adenomatous polyposis coli) mice did not increase inflammation or cause tumorigenesis [84]. *APC* is a tumour suppressor gene that when mutated, is associated with the development of CRC [85]. It was suggested that only certain strains of *F. nucleatum* may harbour carcinogenic properties and that specific interaction with other microbes may be necessary to elicit neoplastic generation. Discrepancies between this research and previous studies were documented by Tomkovich et al. they suggested that strain-specific properties, individual mouse genetics or variations in microbial environments between laboratory facilities may influence study outcomes [84]. Other studies however, infer a causal role for *F. nucleatum* in CRC genesis and advancement [8, 22, 86]. Taken together, these reports emphasise the complexity of the interaction between host and microbial environment and its influence in human disease. However, mounting evidence suggests that *F. nucleatum* may be considered a significant driver in CRC development and progression.

Recent evidence also suggests a potential role for other non-nucleatum Fusobacteria in the development of CRC including *F. hwasookii* [87] and *F. gonidiaformans, F. periodonticum* and *F. varium* [88]. Purcell et al. found *F. hwasookii* to be strongly associated with CRC (particularly in CMS1 (Consensus Molecular typing 1)). It was suggested that given its genetic similarity to *F. nucleatum* and presence of the *fadA* gene, it is possible that *F. hwasookii* could play a similar role in CRC development [87]. Another recent study also revealed that homologues of the FadA adhesion factor were not only present in *F. nucleatum* but also in other Fusobacterium, including *F. varium* and *F. ulcerans*, suggesting a possible link with CRC [88]. This data once again confirmed association between CRC and *F. nucleatum*, which with *F. varium,* was the most significant bacteria identified in the CRC cohorts examined [88].

#### Escherichia coli

*Escherichia coli* resides in the human intestinal microbiome and is considered both a harmless commensal and a potentially dangerous pathogen. Cyclomodulins generated by pathogenic *E. coli* include Colibactin, CDT (Cytolethal Distending Toxin) and CNF (Cytotoxic necrotizing factor) (See Table 1). Cyclomodulins are genotoxic, they dysregulate the cell cycle and are implicated in both cell differentiation and apoptosis, all of which are supportive mechanisms in colonic oncogenesis [17]. Nougayrede et al. identified colibactin in 2006 and subsequent investigations have suggested *E. coli* positive *Pks* (Polyketide synthetase island) strains role in colorectal carcinogenesis, through low grade continuous DNA damage at enterocyte level [89]. Colibactin is a compound synthesised by enzymes encoded by the *Pks* locus, containing up to 18 genes including colibactin A (*clbA*) and colibactin P (*clbP*), suspected to be responsible for virulence (Table 1), [63]. *E. coli* is implicated as a potential player in CRC, particularly colibactin producing strains, verified in both *in vitro* and *in vivo* animal studies [31, 90]. Tomkovich et al. also found that colibactin producing *E. coli* strains instigated CRC progression in murine models [84].

Studies have shown that 86% of *E. coli* strains (B2 phenotypes) isolated from colon adenocarcinoma tissue of 48 human subjects were cyclomodulin positive [91]. Both CDT and colibactin have DNase activity, creating double stranded DNA breaks within epithelial cells [18]. Dalmasso et al. also established that colibactin emitting *E. coli* promote cell proliferation and tumour maturation in CRC. They suggested that this process alters the tumour microenvironment and that several growth factors (HGF (Hepatocyte Growth Factor), FGF (Fibroblast Growth Factor) and GM-CSF (Granulocyte–macrophage-Colony Stimulating Factor)) from senescent cells can be activated to encourage tumour development. This concept was supported by referring to results obtained from both CRC mouse models and human cancer biopsy samples that were infiltrated with *E. coli Pks* strains [5].

A recent review summarised the function of CNF, it is linked to aberrant cell growth and differentiation. CNF activates the G1-S cell cycle transition and so elevates DNA replication. The total number of cells does not increase but rather cells develop into a multinucleated format, thus, this toxin can interfere with cell differentiation and subsequently induce cell death [92]. CNF has a complex function that includes impairment of normal mitotic events, apoptosis and cell survival, linking it to carcinogenesis [93]. CNF may also harbour anti-cancer properties [92]. As noted in a recent critical review, there is a possibility that *E. coli* strains are attracted to the tumour microenvironment as they tend to thrive in this anaerobic niche. Therefore, dispute arises as to whether *E. coli* strains can cause cancer or if they are found in higher quantities in tumour tissue due to their survival instincts [63].

#### Bacteroides fragilis

*Bacteroides fragilis* is another member of the human gut microbiome and is both an important commensal bacterium and well-established pathogen. This species is understood to be a poor prognostic associate of CRC [94]. It is associated with CRC through the secretion of *B. fragilis* toxin (BFT), specifically *Enterotoxigenic B. fragilis* (ETBF) strains. BFT is a zinc-dependent metalloprotease toxin encoded by a *B. fragilis* pathogenicity island (BfPAI) thought to be exclusive to ETBF strains [95]. BFT stimulates colonic Stat3 activity and TH17 mucosal immune response which promotes CRC development in mouse models [13]. Described as an oncogenic bacterial toxin, its action is outlined in Table 1 [13, 64]. Studies have shown a link between ETBF and precancerous and cancerous colorectal lesions [14, 21]. ETBF is also a known factor in both Inflammatory Bowel Disease (IBD) and diarrheal linked illness; furthermore, IBD is a potential risk factor for CRC development [96]. Recent evidence suggests a relationship between *B. fragilis* and CRC but further research is necessary with larger numbers of human subjects to confirm these findings.

### Pancreatic cancer

PC is predominantly associated with poor clinical outcomes. An estimated 85% of cases are correlated to PDAC, with only 24% of PDAC patients surviving the first-year post diagnosis. The 5-year survival rate is estimated between 5 and 7% [97].

*Porphyromonas gingivalis* and *Aggregatibacter actinomycetemcomitans* are linked to an increased risk of PDAC [11]. It was established through a nested case control study of 361 PDAC patients and 371 matched controls, who were observed over a 10-year period, that *P. gingivalis* and *A. actinomycetemcomitans* increase the risk of acquiring the disease by 59% and 50%, respectively [98]. Both oral microbiome organisms are causative agents in periodontitis, which may implicate oral mucosal integrity [99, 100], and studies have proposed that periodontitis is associated with PC pathogenesis [11, 101]. It has been confirmed that oral microbes can spread to pancreatic tissue and other organs through translocation and migration. Their transportation is facilitated via the blood vessel network and/or digestive tract. Once located at distant sites, disruption of the immune system and subsequent inflammation can expediate cancer evolution [102].

*P. gingivalis* is a prominent pathogen with links to orodigestive cancers [103]. It exerts its toxicity through various virulence factors; however, recent studies have highlighted a protein PPAD (Porphyromonas Peptidyl Arginine Deaminase) which until recently was thought to be unique to *P. gingivalis* [104, 105]. However, Gabarrini et al. (2020) reported PPAD homologues in other Porphyromonas species, including *P. gulae* and *P. loveae* [106]. It has been hypothesised that this gene could be implicated in PC tumorigenesis through *P53* activity and *KRAS* (Kirsten-ras) mutation (See Table 2), [65]. *P53* is a tumour suppressor gene, that is usually involved in cell cycle arrest which enables damaged cells to repair their DNA or if damage is irreparable, be targeted for apoptosis [85]. Mutation of *P53* is common in cancer [85], and is mutated in over 50% of all human cancers [107]. Studies highlighted by Ogrendik, noted that *P53* irregularities are prevalent in PC [108, 109]. *KRAS* is an oncogene*,* which normally has GTPase activity, mutations of this gene interfere with GTPase activity and can generate inappropriate cell proliferation and transformation involved in tumorigenesis and poor prognostic outcomes [85]*. KRAS* mutation is related to aggressive disease and poor prognosis in certain pathologies and is understood to be mutated in the majority of PDAC cases [110]. *P. gingivalis and* PPAD have been studied in relation to its implication in rheumatoid arthritis, a condition linked to periodontitis and citrullination. Citrullination is a post translational modification catalysed by the PPAD enzyme. Citrullination by PPAD supports the survival of *P. gingivalis* in infected and inflamed tissue [105]. It is evident that both *in vitro* and *in vivo* studies to further understand the impact of this virulent enzyme on pancreatic tissue and its potential influence in PC are required.

**Table 2:** Summary of bacterial genetic loci with anti-cancer potential as outlined in recent literature

Bacterial Species	Gene Product	Potential action	Potential consequence	References
*Salmonella typhi*	ClyA	Shown to prevent tumour growth when used concurrently with radiotherapy in mice	Potential anti-cancer activity	[25]
*Escherichia coli*	ClyA	Associated with retardation of both primary tumour growth and development of metastasis when used with radiotherapy in mice	Potential anti-cancer activity	[111]
*Campylobacter jejuni*	CDT	Cytotoxic activity potential as an anti-cancer agent	Potential anti-cancer activity	[27, 112]
*Streptococcus pyogenes*	SLO	Can attach to cholesterol in the cell membrane and has an ability to create large pores in the plasma membrane. Cytotoxic activity	Potential anti-cancer activity; Slowed development of tumour growth in metastatic breast cancer	[24, 113]
*Clostridium perfringens*	CPE	Binds directly to receptors claudin-3 and claudin-4, both of which are upregulated in cancer tissue. Mainly colorectal cancer	Potential anti-cancer activity shown in mice colon tumour and gastric cancer cell lines	[26, 114]
*Aggregatibacter actinomycetemcomitans*	LtxA	Induce cell apoptosis in lymphocytes	Potential anti-cancer activity	[115]
*Helicobacter pylori*	NapA	Potent immunomodulator due to induction of Th1 and Th2 response	Potential protective mechanism in relation to cancer	[116]
*Pseudomonas aeruginosa*	PE toxin	Enzymatic action, implicated in cancer cell death in numerous human and murine cancer cell lines	Anti-cancer action	[117–119]

Unsurprisingly, given *H. pylori’s* notoriety relating to GC, recent studies have investigated its potential role in PC. A retrospective study performed by Kumar and colleagues verified *H. pylori*’s relationship with GC, but also focused on its potential correlation with PC. Following analysis of 103,595 patients, they deduced that PC after *H. pylori* infection was rare with an incidence rate of only 0.37% and 0.54% after 5 and 10 years, respectively [120]. Another large cohort prospective study of a Japanese population could not confirm a direct link with the *H. pylori* infection and atrophic gastritis and onset of PC but suggested that a history of atrophic gastritis in smokers, regardless of *H. pylori* status, did increase the risk [121]. Relating this pathogen with PC remains controversial, but some researchers still suggest tenability between the two [122, 123]. These results challenge the possible association with *H. pylori* and PC but once again highlight the need for further investigation in this area.

### Metastatic disease

Metastatic disease relates to cancer cells which have spread to neighbouring tissues and distant organs from the primary tumour site, approximately 90% of cancer deaths are a subsequence of metastatic development [124]. It is unclear if human microbial residents travel alongside cancerous cells to avail of necrotic tumour tissue to colonise and thrive, or if they in fact are a possible causative factor in tumorigenesis and metastatic growth. Recent studies suggest the latter [19, 125].

The *B. fragilis* BFT is a prospective instigator in secondary tumour growth (See Table 1). Its activity leads to vulnerability in epithelial tight junctions and a reduction in cellular adhesion, creating an opportunity for tumour cells to relocate to distant tissue and metastasise in other organs [51]. Bonnet et al. demonstrated a strong link between poor prognostic outcomes for CRC with pathogenic *E. coli* strains, with these strains being more prevalent in the mucosal tissue samples of patients with advanced stages of the disease (Stages III and IV) [18]. *P. gingivalis* produces another virulent protein; Gingipains. Gingipains interacts with protease activated receptor 2 (PAR2) promoting activation of the MMP-9 (Matrix Metalloprotease-9) proenzyme into its mature active form. MMP-9 promotes the destruction of basement membrane and extracellular matrix, an action associated with tumour cell and secondary tumour maturation. This process may correlate to oral cavity metastasis [66]. *H. pylori* CagA positive strains, may also be implicated in promoting metastatic development. It has been suggested that these strains are involved in cellular remodelling, angiogenesis, invasion of tumour cells and ultimately metastatic formation [126]. Literature documenting this is however limited.

Furthermore, *F. nucleatum* when attached to E-cadherin cells expressing the virulence factor FadA may increase the risk linked to secondary tumour progression associated with CRC [127]. Xavier et al. (2020) also proposed that *F. nucleatum* might be transported alongside primary cancerous cells to other organs. This process has been correlated to liver secondaries, more aggressive disease progression and poor outcomes for CRC patients [128]. Recent studies have also highlighted *F. nucleatums* connection with metastatic disease and poor prognostic outcomes [19, 129–131]. Bullmann et al. found an association with distant metastases and primary CRC, and have also detected *F. nucleatum* in liver metastases [19]. Chen and colleagues established that *F. nucleatum* was present in abundance in CRC samples from patients with metastasis. *F. nucleatum* was detected in 75.81% of metastatic CRC samples, in comparison to 43.75% found in the non-metastatic CRC tissues [129]. Subsequent studies on *F. nucleatum* infected mouse models showed pro-metastatic activity linked to increased cell mobility and upregulation of CARD3 (Caspase Activation and Recruitment Domain 3) associated with inflammatory and immune responses [129]. Another study demonstrated that exosomes secreted by *F. nucleatum* infected cells were shown to promote CRC metastasis [130]. Salvucci et al. identified a patient subset (CMS4/CRIS-B (Consensus Molecular Typing 4/Colorectal Cancer Intrinsic subtyping)) as having poorer clinical outcomes when their CRC tumours displayed mesenchymal traits and were associated with higher prevalence of *F. nucleatum* and fusobacteriales [131].

## Microbial cytotoxic genes with therapeutic potential

Microbial genes that produce cytotoxic products are a topic of interest in recent scientific literature [75, 132]. The term cytotoxic refers to cell killing or toxicity leading to the destruction of cells, and virulent genes present in certain bacteria are known to possess potent lethal capabilities [25, 114]. The possibility of manipulating the microbiome/microbiota for therapeutic advances in oncology is currently under investigation due to such potential cytotoxic action on tumour cells. Cytotoxic products have certainly demonstrated possible carcinogenic activity [133] but others have also shown anti-carcinogenic potential [132] which will be the main focus of this section (See Table 2).

### Cytolysin A

Cytolysin A (ClyA) is a pore-forming cytotoxin [134], suggested as an agent possessing anti-cancer properties, primarily secreted by *Escherichia coli* and *Staphylococcus aureus* strains. It exerts a permeating function, by creating multimeric pores and imposing cell death in the eukaryotic membrane by the caspase-dependent pathway [135].

A study performed on CT26 colon cancer BALB/c mice demonstrated that *S. typhi* strains producing the cytotoxic protein ClyA can reduce tumour growth when used concurrently with a cumulative dose of 21 Gy (Gray) of radiotherapy, which is lower than traditional treatment doses of 65–70 Gy [25]. This study demonstrated a reduction in the volume of the tumour mass in the infected mice on day 40 of the experiment when compared with the control group, 200mm^3^ and ≥ 1,200mm^3^, respectively. However, tumour tissue was not completely eliminated [25]. Another study also investigated BALB/c mice infected with CT26 colon cancer cells; to induce development of primary cancer, and subsequently lung metastasis [111]. An engineered *E. coli* strain K12, with the ability to secrete ClyA was used as a treatment alongside radiation therapy (a cumulative dose of 21 Gy). Dramatic effects were observed on reduction of primary tumour growth and in suppressing the development of lung metastasis, total elimination of the tumours was also documented [111]. This approach could potentially significantly reduce the side effects associated with traditional radiotherapy treatment. It is evident however, that human trials are lacking at present.

### Cytolethal distending toxin

The Cytolethal distending toxin (CDT) has been mentioned above as a product of pathogenic *E. coli* strains. Numerous other gram-negative bacteria found within the human gastrointestinal tract microbiome are also known to produce this toxin*.* These include *A. actinomycetemcomitans, Campylobacter spp.* and *Helicobacter spp.* CDT has 3 subunits encoded by the genes *cdtA, cdtB* and *cdtC*, with *cdtB* responsible for toxicity [136]. *S. typhi* also produces CDT. However, it differs from the other bacterial species in that it possesses a *cdtB* gene but does not encode *cdtA* or *cdtC* [137]. CDT has been described as both a genotoxin and cyclomodulin, and is a potential causal factor in tumorigenesis. Genotoxic action involves DNase activity which creates DNA double stranded breaks, leading to cell cycle arrest and cytotoxicity [136].

*Campylobacter jejuni* also produces this genotoxin. An investigation by He and colleagues (2019) revealed that a specific strain of this microbe, human clinical isolate *C. jejuni* 18–176 was linked to colorectal tumorigenesis [133]. They also noted alterations to the microbial composition and transcriptomic responses which is determined by the subunit of CDT (*cdtB*) activity. This study showed that mice infected with this particular strain of *C. jejuni* developed a significantly higher number of tumours than those not infected, 10.7 versus 4.0, and a substantially higher level of larger tumours with a diameter measuring ≥ 3 mm; 77% (54/75) in the infected group in comparison to 43% (9/21) in the control group. They proposed that this was the first study to link *C. jejuni* to CRC development [133]. Identifying its impact in CRC, highlights this toxin as a potential therapeutic target.

Despite its ability to induce carcinogenesis through its genotoxic activity, there is also evidence given its cytotoxic nature in various cancer cell lines and cancer mouse models for a potential use for CDT as an anti-cancer agent [27, 112]. Bachran et al. developed a fusion protein, which enabled transportation of CdtB from *Haemophillus ducreyi* into the cytosol of tumour cells of Lewis Lung cancer infected mice via a delivery system containing modified anthrax toxin. Subsequent inhibition of tumour growth leading to a 90% cure rate was documented with low toxicity observed in the mice studied [112]. Cytotoxic activity was also documented in a number of tumour cell lines tested in this study, including Hela cells (human cervical carcinoma cell line) and HN6 cells (human head and neck cancer cell line) [112]. Wang et al. documented anti-tumour activity and prolonged survival when *Salmonella* carrying CdtB therapy was used in mice with established melanoma and breast tumours [27]. However, further studies are required to validate CDT as a potential anti-cancer therapy, particularly in human subjects.

### Streptolysin O

*Streptococcus pyogenes*, a gram-positive, human-specific pathogen, secretes the protein Streptolysin O (SLO) which is implicated in cytolysis and apoptosis [24]. SLO is a cholesterol-dependent cytolysin that is secreted by most strains of β-hemolytic streptococci and is encoded by the *slo* gene, which is part of a 3 gene operon [138]. SLO activity is outlined in Table 2. A 2006 study showed it to induce anti-tumour activity in cell culture including 293 T cells (HEK 293 cell derivatives), A549 cells (human lung cancer cells) and U343 cells (Human glioma cells); and C33A (human cervical cancer cells) xenograft nude murine models [24]. SLO has also showed promise *in vitro* in inhibiting the progression of metastatic BC [113].

### Clostridium Perfringens enterotoxin

*Clostridium perfringens* is an anaerobic pathogenic microbe linked primarily to food poisoning which secretes CPE (*Clostridium perfringens* enterotoxin) [26]. It binds directly to receptors claudin-3 and claudin-4 (Table 2), both of which are upregulated in cancer tissue, particularly in CRC, BC, ovarian cancer and GC [26, 114]. CPEs action in relation to these receptors can notably reduce tumour proliferation. Pahle et al. investigated CPE suicide gene therapy and proved that it led to rapid and effective colon tumour cell death both *in vitro* and *in vivo* [26]. Liang and colleagues demonstrated that claudin-4 mediated toxicity induced cytotoxicity in human GC cells (SGC7901) and inhibited tumour growth in SGC7901 xenografts in a nude mouse model. They did note possible safety concerns relating to CPE therapy: skin necrosis at the injection site, gastrointestinal inflammation and tissue damage [114]. Further studies both *in vivo* and *in vitro* are clearly needed before investigating its clinical use as a potential treatment.

### Leukotoxin and Cytotoxin associated Gene A

*A. actinomycetemcomitans* is another potentially aggressive human oral commensal, known to express various genes with cytolethal capabilities, such as *cdT, ltxA* (Leukotoxin A) and *cagE* (Cytotoxin associated gene A), all of which contribute to its virulence. It is the only oral pathogen that generates both the protein exotoxins (CDT and LtxA) [115].

LtxA is potentially its most influential toxin. Its operon belongs to the core genome of *A. actinomycetemcomitans* and is believed to be present in all investigated strains of the bacterium [139]. It can impact leukocytic cells with varying strategies towards lethality. By targeting a specific cell receptor, and initiating neutrophil degranulation, it causes extensive lysosomal enzyme activation, net-like structure formation and MMPs activity, subsequently inducing cell death in lymphocytes [115]. Properties such as these demonstrate how certain commensal bacteria with cytotoxic gene activity have been linked to oncological therapeutics.

### Probiotic strains showing anti-cancer properties

The term probiotic was defined in 2001 by the Food and Agricultural Organisation of the United Nations and the World Health Organisation, the definition was reinforced in 2014 by a panel of experts as ‘live microorganisms which when administered in adequate amounts confer a health benefit on the host’ [140]. Probiotic strains most commonly belong to Lactic acid bacteria (LAB), and include both *Lactobacillus* and *Bifidobacterium* species [141]. Benefits such as immune system support and the restoration of gut microbial imbalance following antibiotic treatment have been identified. There is some concern however amongst the scientific community relating to the ability of probiotic strains to survive harsh intestinal acidic or alkaline conditions, their potential involvement in resistant gene transfer, and infection risk in their use as therapies in immunocompromised individuals [142]. However, recent studies have identified and investigated probiotic strains of bacteria which may have a potential role in anti-cancer activity [141, 143, 144].

Parisa and colleagues, found that a Bifidobacteria cocktail of five strains, including *B. bifidum*, *B. breve* and *B. longum* species, showed significant anti-carcinogenic impact including tumoricidal activity and down regulation of EGFR (Epidermal Growth Factor Receptor), HER2 (Human epidermal growth factor receptor 2) and Cox-2 (Cyclooxygenase-2). EGFR, HER2 and Cox-2, when up-regulated have all been associated with CRC advancement, including inappropriate cell-proliferation and metastatic development [141]. Considerable anti-cancer effects were documented in both LS174T (human colon adenocarcinoma cells); 20.5% primary cell apoptosis when compared to IEC-18 (normal cells) with approximately 3% cell apoptosis following a 120-h incubation period with the bacterial strains used in this study. They also noted anti-tumour activity in CRC mouse models when treated with the bifidobacterial cocktail, which included an improvement in the Disease Activity Index (DAI), restoration of colon length and reduction in tumour numbers when compared to untreated mice. Treatment also prevented the progression of the disease to more advanced stages. These researchers suggest that the bifidobacterial cocktail investigated in this study could be considered as a nutritional supplement for concurrent use with the suggested chemotherapy drugs (Cetuximab and Trastuzumab) to potentially treat and prevent CRC [141]. Another recent study demonstrated significant human colon cancer cell line (HT-29 and Caco-2) apoptosis with Bifidobacterial strains. In HT-29 cells the highest apoptotic activity was seen with *B. bifidum* (53.32%) and in Caco-2 cells the most remarkable cytotoxicity was noted with *B. bifidum* (79.8%), *B. animalis subsp. lactis* (68.07%) and *B. animalis subsp. animalis* (68.3%) [145]. In a recent review, an advantage of Bifidobacterial strains as potential vectors for novel anti-cancer therapies was suggested due to their anaerobic and non-pathogenic nature, this species would have the ability to grow in necrotic tumour tissue while not posing an infectious threat to potentially vulnerable patients receiving bacterial-based treatments [146].

The potential for *Lactobacillus* based probiotic treatments in the suppression of cervical cancer has also been investigated [147]. *Lactobacillus* produces various metabolites such as exopolysaccharides and peptidoglycans which potentially prevent tumour cell proliferation [147]. This has been noted as a protective measure by this species as part of the resident flora of the vagina [147]. Chuah et al. deduced that post metabolites produced by six different strains of *Lactobacillus plantarum* showed selective cytotoxicity through the prevention of cell proliferation and initiation of cell death against various cancer cells including breast, colorectal, cervical and leukemic. It was reported that normal cells remained intact during the experiments conducted [143]. Park et al. demonstrated anti-cancer effects including apoptosis in A375 human malignant melanoma cells when treated with *L. plantarum* L-14 extract [144]. These results show promise for the use of *L. plantarum* post metabolites as part of a cancer therapy regime.

In another recent study, aerosolized probiotic and antibiotic treatment showed promise in the treatment of lung cancer infected mice and in prevention of more advanced disease [148]. Le Noci et al. demonstrated that mice treated with aerosolized Vancomycin (directed at gram-positive bacteria) and Neomycin (directed at gram-negative bacteria) showed a statistically significant reduction in commensal bacterial load, which correlated to decreased regulatory T-cells and enhanced T-cell and Natural Killer cell activation, leading to a significant reduction in melanoma B16 lung metastases. Furthermore, *Lactobacillus rhamonosus* administered in an aerosolized form enhanced immunity against B16 lung metastases. The delivery of Probiotics (including strain *B. bifidum* MIMBb23sg) and antibiotics was also shown to improve chemotherapeutic activity against B16 metastases [148].

Notably, probiotic species possibly have an advantage in oncological treatment as they are deemed generally safe, cheap and readily available, and have also shown limited impact on normal cells in research to date. However, the concerns previously outlined acknowledge that further clinical studies are vital, particularly involving human subjects.

## Recent advances in potential cytotoxic gene therapies

Recent literature has proposed the use of bacterial toxic gene products as anti-tumour agents (See Table 2). Various delivery strategies have been developed to target tumour tissue. These include attenuation of bacteria in the development of vaccines, genetic modification strategies in the development of bacterial vectors and development of Antibody Drug Conjugates (ADC’s), as will be discussed in Sections Attenuated bacterial vaccines and bacterial vectors and Immunotoxins in targeted cancer therapy.

As highlighted in this review, it is well documented that bacteria have been detected in tumour tissue and that necrotic tumour tissue potentially provides a perfect habitat for anaerobic bacteria such as *Clostridium spp*. and *Bacillus spp.* [149]. In a recent publication, resident gut bacteria including *Clostridium spp., Bifidobacterium spp., Salmonella typhimurium, Vibrio cholera, Listeria monocytogenes* and *Bacillus spp.* were listed as the most prevalent species of bacteria found within the tumour microenvironment [150]. The tumour microenvironment is formed by the tumour itself and aids tumour survival, it consists of proliferating tumour cells, stroma, blood vessels, inflammatory cells and other correlated tissues [151]. Studies have shown that species such as *Salmonella spp*., *Listeria spp*., *Clostridium spp.*, and *E. coli* may have anti-tumour activity and potential possession of therapeutic capabilities [152–155]. These bacteria may have the capability of continuously secreting cytotoxic products once present within a malignant location, achieved through GM (Genetic Modification) and delivery into necrotic tissue via anti-tumour vectors, as described below. This characteristic would be advantageous over traditional oncology therapeutics which often have difficulty penetrating deep into tumour tissue, but tend instead to act on proliferating cells [156].

### Attenuated bacterial vaccines and bacterial vectors

Bacterial strains are attenuated for therapeutic cancer treatment to reduce the virulence of potential pathogens and reduce the impact on the immune response of the host [157]. Genetically engineered bacteria may include the use of bacterial vectors to allow the delivery of therapeutic agents directly to tumour tissue, through for example the use of plasmids or allow the expression of a particular gene that may have therapeutic value [149]. *Salmonella spp.* has shown promise as a potential cancer treatment; it was initially studied as an anti-tumour vector in melanoma mouse models with evidence of anti-tumour activity [157]. Various GM forms of *Salmonella typhi* have been investigated. Attenuated strain *Salmonella typhimurium*, VNP20009, has proven to have an impressive safety record [158]. It is GM through deletion of the *purL* gene (allowing genetic stability), a deletion of the *msbB* gene (to limit septic shock complications) and antibiotic susceptibility. A study demonstrating its safety and its anti-tumour effect in both mice and monkey tumour models found it would potentially be safe for human administration [158]. Although, a human Phase 1 clinical trial was conducted, involving 24 metastatic melanoma patients and 1 metastatic renal cell carcinoma patient, tumour elimination or impact on tumour growth was not observed. Safety for human use was however confirmed [159]. More recently, VNP20009 strain was shown to induce apoptosis in various leukaemia cell lines *in vitro* and *in vivo.* Significant inhibition in the proliferation of MLL-Af9-induced acute myeloid leukaemia (AML) cells (inhibition rate was 82.82%) and increased survival of AML mice was documented [160]. In 2015 another GM strain, *S. typhi A1-R* showed anti-tumour activity in Lewis lung carcinoma mouse models [161]. *S. typhi* A1-R is GM to prevent impact to normal tissue and has also been shown to be effective against tumour tissue in human pancreatic cancer cell lines and sarcoma mouse models [162, 163]. Positive results were also seen when *S. typhi* A1-R was used in combination with chemotherapy drugs; this concurrent treatment promoted targeting of *S. typhi* A1-R in melanoma PDOX (Patient-derived orthotopic xenograft) mouse models; this melanoma was previously resistant to chemotherapy treatment [164]. Studies are ongoing to investigate the use of *Salmonella* as an anti-tumour therapy [163, 165]. *Salmonella*-based therapies have proven to specifically target and penetrate tumour tissue. Anti-tumour effects have been observed in numerous *in vivo* and *in vitro* studies and human tolerance has been recorded. These findings suggest GM Salmonella strains deserve further investigation, perhaps with a focus on effective concurrent treatments which may increase the effectiveness of chemotherapeutics.

*Clostridium* is another genus within the human microbiome in which certain strains have exhibited anti-cancer potential [154, 166]. As a strict anaerobe it can thrive in anaerobic necrotic tumour environments; viewed as one of the most promising anti-oncolytic benefits of anaerobic bacterial strains. More specifically, *Clostridium noyvi* has exhibited powerful cytotoxic effects on tumour cells. *Clostridium noyvi-NT* is a GM therapy and attenuated strain (therefore avoiding systemic toxicity) developed based on these abilities and has undergone trials in the treatment of solid tumours including soft tissue sarcoma, osteosarcoma, malignant melanoma and mast cell tumours in dogs, and leiomyosarcoma in a human patient [154]. A recent phase 1 clinical trial with 24 human subjects with advanced solid tumours showed promising results on receiving intra-tumoral injection of *Clostridium* noyvi-NT spores. A total of 9 patients (41%) showed a decrease in the size of the injected tumour mass while disease progression stabilised in 19 patients (86%) [166].

Neutrophil activating protein (NapA), another virulence factor of the *H. pylori* species, has been shown to have a potential protective mechanism in relation to cancer, as outlined in Table 2 [116]. In a recent study, a GM *L. lactis* strain incorporating the *napA* gene showed promise in the form of an oral vaccine as an anti-*H. pylori* candidate in relation to GC. It was shown to induce an immune protective response in mice. It is still unclear as to whether NapA protein would reduce gastrointestinal inflammation or if it had potential to increase it and so requires further examination. Interestingly, NapA has exhibited the ability to cause necrosis in bladder tumours in murine models in previous studies [116].

### Immunotoxins in targeted cancer therapy

Immunotoxins are cytotoxic fusion proteins that involve the conjugation of an antibody fragment that binds to a cancer cell and a protein toxin fragment that causes cell apoptosis, also referred to as ADC’s [167]. *Pseudomonas aeruginosa* is known to produce the virulent Pseudomonas exotoxin A (PE toxin), believed to have an enzymatic function [117]. This toxin has been implicated in cancer cell death in various murine and human cancer cell lines [118, 119]. It has been utilised to create a number of immunotoxin treatments, with encouraging pre-clinical and phase I, II and III clinical test outcomes [167, 168]. However, there has been issues over the years identified with regards to resistance to treatments linked to potential PE-based treatments, but ongoing research appears to be addressing this problem. These are discussed in detail in recent publications [167, 168].

In 2018 a PE toxin derived drug called Lumoxiti was approved by the US Food and Drug Administration aimed at treating hairy cell leukaemia. This monoclonal antibody drug comprises an anti-CD22 fv and a 38 kDa portion of Pseudomonas exotoxin A [167]. Release of such therapies onto the market emphasise the possibilities relating to various bacterial strains and the cytotoxic secretions they produce in the development of novel targeted therapies. Another recombinant therapy that incorporates PE; LMB-100 has also undergone Phase I/II clinical trials in 24 patients with advanced PDAC in combination with nab-Paclitaxel. Although anti-tumour activity was documented during this trial this combination treatment proved too toxic for the patients treated. Further clinical trials testing LMB-100 in combination with other drugs are planned [169]. It is evident that ongoing animal studies and human clinical trials are needed to address safety issues with such treatments.

It is suggested that concurrent treatments may be required alongside such novel bacterial-related therapies as these species may not completely eradicate the malignant tissue. Bacterial treatments may act as sensitising agents for chemotherapy [170]. These authors also referred to the detrimental potential in using bacterial therapies in already immunocompromised hosts [170]. It is acknowledged that cancer patients are vulnerable and prime candidates for opportunistic infection. Some of the issues identified with administration of bacterial therapies, include DNA mutations causing changes that could impact benefit of treatment or create overwhelming infection. Recombinant DNA mechanisms have largely accounted for such concerns, but ongoing research and developments are necessary to ensure utmost safety moving forward.

## Conclusion

In summary, the human microbiome and its potential link to cancer has prompted great scientific interest in recent years. Although only one bacterium (*H. pylori*) has been established to have a direct causal role in cancer, strong evidence indicates the involvement of others, specifically *F. nucleatum in* CRC and *P. gingivalis* in PC. Both are deserving of further investigation. As highlighted in this review, microbial genetic determinants associated with certain bacterial species could potentially provide vital clues in the causation of various oncological pathologies and thus lead to the identification of appropriate oncological biomarkers and therapeutic targets. The cytotoxic activity of certain species, as outlined previously have shown hope for the future development of novel therapeutics for cancers previously associated with poor prognosis, such as PC. Nevertheless, in 2019 the International Cancer Microbiome Consortium announced limited correlation between commensal bacteria and cancer generation. The panel did accept the undoubted role of *H. pylori* in gastric cancer (GC) but suggested that a causal link between the microbiome and cancer remains somewhat hypothetical and requires further investigation [171]. This statement underlines the requirement for ongoing and novel research to establish the potential link between the microbiome and cancer, and the future development of novel more efficacious treatment strategies.

## Data Availability

Not applicable.

## Abbreviations

CRC: Colorectal Cancer
PC: Pancreatic Cancer
GC: Gastric Cancer
PDAC: Pancreatic Ductal Adenocarcinoma
BC: Breast Cancer
CagA: Cytotoxic-associated gene A
VacA: Vacuolating cytotoxin A
OipA: Outer Inflammatory Protein A
FadA: Fusobacterium adhesion A
Fap2: Fibroblast activation protein 2
RadD: Radiation gene
APC: Adenomatous polyposis coli
CDT: Cytolethal distending toxin
CNF: Cytotoxic necrotizing factor
HGF: Hepatocyte Growth Factor
FGF: Fibroblast Growth Factor
BFT: Bacteroides Fragilis Toxin
PPAD: Porphyromonas Peptidyl Arginine Deaminase
MMP: Matrix Metalloprotease
CARD3: Caspase Activation and Recruitment Domain 3
GM: Genetically Modified
ClyA: Cytolysin A
SLO: Streptolysin O
CPE: Clostridium Perfringens Enterotoxin
LtxA: Leukotoxin A
CagE: Cytotoxin associated gene A
EGFR: Epidermal Growth Factor
HER2: Human Epidermal Growth Factor Receptor 2
Cox-2: Cyclooxygenase-2
IEC-18: Rat normal non-transformed intestinal cell line
LAB: Lactic Acid Bacteria
PDOX: Patient-derived orthotopic xenograft
NapA: Neutrophil activating protein A
PE toxin: Pseudomonas Exotoxin A

## References

[1] Sung H, Ferlay J, Siegel RL, LaversanneSoerjomataram MI, Jemal A (2021). Global cancer statistics 2020: GLOBOCAN estimates of incidence and mortality worldwide for 36 cancers in 185 countries. CA Cancer J Clin.

[2] Sauer AG, Siegel RL, Jemal A, Fedewa SA (2019). Current prevalence of major cancer risk factors and screening test use in the United States: Disparities by education and race/ethnicity. Cancer Epidemiol Biomarkers Prev.

[3] de Martel C, Georges D, Bray F, Ferlay J, Clifford GM (2020). Global burden of cancer attributable to infections in 2018: a worldwide incidence analysis. Lancet Glob Heal.

[4] Pagano JS, Blaser M, Buendia MA, Damania B, Khalili K, Raab-Traub N (2004). Infectious agents and cancer: Criteria for a causal relation. Semin Cancer Biol.

[5] Dalmasso G, Cougnoux A, Delmas J, Darfeuille-Michaud A, Bonnet R (2015). The bacterial genotoxin colibactin promotes colon tumor growth by modifying the tumor microenvironment. Gut Microbes.

[6] Howley PM (2015). Gordon Wilson Lecture: Infectious Disease Causes of Cancer: Opportunities for Prevention and Treatment. Trans Am Clin Climatol Assoc.

[7] Mégraud F (2005). A humble bacterium sweeps this year’s Nobel Prize. Cell.

[8] Castellarin M, Warren RL, Freeman JD, Dreolini L, Krzywinski M, Strauss J (2012). Fusobacterium nucleatum infection is prevalent in human colorectal carcinoma. Genome Res.

[9] Kostic AD, Gevers D, Pedamallu CS, Michaud M, Duke F, Earl AM (2012). Genomic analysis identifies association of Fusobacterium with colorectal carcinoma. Genome Res.

[10] Abed J, Maalouf N, Parhi L, Chaushu S, Mandelboim O, Bachrach G (2017). Tumor targeting by Fusobacterium nucleatum: A Pilot Study and future Perspectives. Front Cell Infect Microbiol.

[11] Fan X, Alekseyenko AV, Wu J, Peters BA, Jacobs EJ, Gapstur SM (2018). Human oral microbiome and prospective risk for pancreatic cancer: A population-based nested case-control study. Gut.

[12] Inaba H, Sugita H, Kuboniwa M, Iwai S, Hamada M, Noda T (2014). Porphyromonas gingivalis promotes invasion of oral squamous cell carcinoma through induction of proMMP9 and its activation. Cell Microbiol.

[13] Wu S, Rhee KJ, Albesiano E, Rabizadeh S, Wu X, Yen HR (2009). A human colonic commensal promotes colon tumorigenesis via activation of T helper type 17 T cell responses. Nat Med.

[14] Zamani S, Taslimi R, Sarabi A, Jasemi S, Sechi LA, Feizabadi MM (2020). Enterotoxigenic Bacteroides fragilis: A Possible Etiological Candidate for Bacterially-Induced Colorectal Precancerous and Cancerous Lesions. Front Cell Infect Microbiol.

[15] Ho JTK, Chan GCF, Li JCB (2015). Systemic effects of gut microbiota and its relationship with disease and modulation. BMC Immunol.

[16] Gensollen T, Iyer SS, Kasper DL, Blumberg RS (2016). How colonization by microbiota in early life shapes the immune system. Science (80- ).

[17] Buc E, Dubois D, Sauvanet P, Raisch J, Delmas J, Darfeuille-Michaud A (2013). High Prevalence of Mucosa-Associated E coli Producing Cyclomodulin and Genotoxin in Colon Cancer. PLoS One.

[18] Bonnet M, Buc E, Sauvanet P,  Darcha C, Dubois D, Pereira B (2014). Colonization of the human gut by E. coli and colorectal cancer risk. Clin Cancer Res.

[19] Bullman S, Pedamallu CS, Sicinska E, Clancy TE, Zhang X, Cai D (2017). Analysis of Fusobacterium persistence and antibiotic response in colorectal cancer. Science (80- ).

[20] Bakhti SZ, Latifi-Navid S, Zahri S (2019). Unique constellations of five polymorphic sites of Helicobacter pylori vacA and cagA status associated with risk of gastric cancer. Infect Genet Evol.

[21] Purcell RV, Pearson J, Aitchison A, Dixon L, Frizelle FA, Keenan JI (2017). Colonization with enterotoxigenic Bacteroides fragilis is associated with early-stage colorectal neoplasia. PLoS ONE.

[22] Yang Y, Weng W, Peng J, Hong L, Yang L, Toiyama Y (2017). Fusobacterium nucleatum Increases Proliferation of Colorectal Cancer Cells and Tumor Development in Mice by Activating TLR4 Signaling to NFκB, Upregulating Expression of microRNA-21. Gastroenterology.

[23] Chang WL, Yeh YC, Sheu BS (2018). The impacts of H. pylori virulence factors on the development of gastroduodenal diseases. J Biomed Sci.

[24] Yang WS, Park SO, Yoon AR, Yoo JY, Kim MK, Yun CO (2006). Suicide cancer gene therapy using pore-forming toxin, streptolysin O. Mol Cancer Ther.

[25] Liu X, Jiang S, Piao L, Yuan F (2016). Radiotherapy combined with an engineered Salmonella typhimurium inhibits tumor growth in a mouse model of colon cancer. Exp Anim.

[26] Pahle J, Menzel L, Niesler N, Kobelt D, Aumann J, Rivera M (2017). Rapid eradication of colon carcinoma by Clostridium perfringens Enterotoxin suicidal gene therapy. BMC Cancer.

[27] Wang WK, Chiang WC, Lai CH, Lee CH (2018). Salmonella-Mediated Cytolethal Distending Toxin Transfer Inhibits Tumor Growth. Hum Gene Ther.

[28] Qin J, Li R, Raes J, Arumugam M, Burgdorf KS, Manichanh C (2010). A human gut microbial gene catalogue established by metagenomic sequencing. Nature.

[29] Forster SC, Kumar N, Anonye BO, Almeida A, Viciani E, Stares MD (2019). A human gut bacterial genome and culture collection for improved metagenomic analyses. Nat Biotechnol.

[30] Dethlefsen L, Huse S, Sogin M L, Relman D.A (2008). The Pervasive Effects of an antibiotic on the Human Gut Microbiota, as Revealed by Deep 16S rRNA Sequencing. Plos Biol.

[31] Arthur JC, Perez-chanona E, Mühlbauer M, Tomkovich S, Uronis JM, Fan T (2013). Intestinal inflammation targets cancer-inducing activity of the microbiota. Science.

[32] Ahn J, Sinha R, Pei Z, Dominianni C, Wu J, Shi J (2013). Human gut microbiome and risk for colorectal cancer. J Natl Cancer Inst.

[33] Hibberd AA, Lyra A, Ouwehand AC, Rolny P, Lindegren H, Cedgård L (2017). Intestinal microbiota is altered in patients with colon cancer and modified by probiotic intervention. BMJ Open Gastroenterol.

[34] Pushalkar S, Hundeyin M, Daley D, Zambirinis CP, Kurz E, Mishra A (2018). The pancreatic cancer microbiome promotes oncogenesis by induction of innate and adaptive immune suppression. Cancer Discov.

[35] Nejman D, Livayatan I, Fuks G, Gavert N, Zwang Y, Geller LT (2020). The human microbiome is composed of tumour type-specific intracellular bacteria. Science.

[36] Wei MY, Shi S, Liang C, Meng QC, Hua J, Zhang YY (2019). The microbiota and microbiome in pancreatic cancer: More influential than expected. Mol Cancer.

[37] Caselli E, Soffritti I, D’Accolti M, Piva I, Greco P, Bonaccorsi G (2019). Atopobium vaginae and Porphyromonas somerae induce proinflammatory cytokines expression in endometrial cells: A possible implication for endometrial cancer?. Cancer Manag Res.

[38] Hieken TJ, Chen J, Hoskin TL, Walther-Antonio M, Johnson S, Ramaker S (2016). The microbiome of aseptically collected human breast tissue in benign and malignant disease. Sci Rep.

[39] Meng S, Chen B, Yang J, Wang J, Zhu D, Meng Q (2018). Study of microbiomes in aseptically collected samples of human breast tissue using needle biopsy and the potential role of in situ tissue microbiomes for promoting malignancy. Front Oncol.

[40] Fernández MF, Reina-Pérez I, Astorga JM, Rodríguez-Carrillo A, Plaza-Díaz J, Fontana L (2018). Breast cancer and its relationship with the microbiota. Int J Environ Res Public Health.

[41] Hilty M, Burke C, Pedro H, Cardenas P, Bush A, Bossley C (2010). Disordered microbial communities in asthmatic airways. PLoS One.

[42] Segal LN, Clemente JC, Tsay J-CJ, Koralov SB, Keller BC, Wu BG (2016). Enrichment of the lung microbiome with oral taxa is associated with lung inflammation of a Th17 phenotype. Nat Microbiol.

[43] Kim HJ, Kim YS, Kim KH, Choi JP, Kim YK, Yun S (2017). The microbiome of the lung and its extracellular vesicles in nonsmokers, healthy smokers and COPD patients. Exp Mol Med.

[44] Lee SH, Sung JY, Yong D, Kim SY, Song JH, Chung KS (2016). Characterisation of microbiome in bronchoalveolar lavage fluid of patients with lung cancer comparing with benign mass like lesions. Lung Cancer.

[45] Jin C, Lagoudas G, Zhao C, Bullmann S, Bhutkar A, Hu B (2019). Commensal Microbiota Promote Lung Cancer Development via γδ T Cells. Cell.

[46] Thomas RM, Jobin C (2016). Is the oncobiome mirage real. Trends Cancer.

[47] Kiss B, Mikó E, Seb˝O É, Toth J, Ujlaki G, Szabó J (2020). Oncobiosis and microbial metabolite signaling in pancreatic adenocarcinoma. Cancers (Basel).

[48] Gori S, Inno A, Belluomini L, Bocus P, Bisoffi Z, Russo A (2019). Gut microbiota and cancer: How gut microbiota modulates activity, efficacy and toxicity of antitumoral therapy. Crit Rev Oncol Hematol.

[49] Sun Z, Xiong CL, Teh SW, Lim JCW, Kumar S, Thilakavathy K (2019). Mechanisms of Oral Bacterial Virulence Factors in Pancreatic Cancer. Front Cell Infect Microbiol.

[50] Lawrence GW, Begley M, Cotter PD, Guinane CM (2020). Potential use of biotherapeutic bacteria to target colorectal cancer-associated taxa. Int J Mol Sci.

[51] Cheng WT, Kantilal HK, Davamani F (2020). The mechanism of bacteroides fragilis toxin contributes to colon cancer formation. Malaysian J Med Sci.

[52] Goodman B, Gardner H (2018). The microbiome and cancer. J Pathol.

[53] Kipanyula MJ, Seke Etet PF, Vecchio L, Farahna M, Nukenine EN, Nwabo Kamdje AH (2013). Signaling pathways bridging microbial-triggered inflammation and cancer. Cell Signal.

[54] Herrero R, Park JY, Forman D (2014). The fight against gastric cancer - The IARC Working Group report. Best Pract Res Clin Gastroenterol.

[55] Neal JT, Peterson TS, Kent ML, Guillemin KH (2013). pylori virulence factor CagA increases intestinal cell proliferation by Wnt pathway activation in a transgenic zebrafish model. DMM Dis Model Mech.

[56] Censini S, Lange C, Xiang Z, Crabtree JE, Ghiara P, Borodovsky M (1996). Cag, a pathogenicity island of Helicobacter pylori, encodes type I-specific and disease-associated virulence factors. Proc Natl Acad Sci U S A.

[57] Yamazaki S, Yamakawa A, Okuda T, Ohtani M, Suto H, Ito Y (2005). Distinct diversity of vacA, cagA, and cagE genes of Helicobacter pylori associated with peptic ulcer in Japan. J Clin Microbiol.

[58] Enroth H, Kraaz W, Engstrand L, Nyren O, Rohan T (2000). Helicobacter pylori strain types and risk of gastric cancer: A case-control study. Cancer Epidemiol Biomarkers Prev.

[59] Franco AT, Johnston E, Krishna U, Yamaoka Y, Israel DA, Nagy TA (2008). Regulation of gastric carcinogenesis by Helicobacter pylori virulence factors. Cancer Res.

[60] Yong X, Tang B, Li BS, Xie R, Hu CJ, Luo G (2015). Helicobacter pylori virulence factor CagA promotes tumorigenesis of gastric cancer via multiple signaling pathways. Cell Commun Signal.

[61] Teymournejad O, Mobarez AM, Hassan ZM, Talebi Bezmin Abadi A (2017). Binding of the Helicobacter pylori OipA causes apoptosis of host cells via modulation of Bax/Bcl-2 levels. Sci Rep.

[62] Rubinstein MR, Wang Y, Liu W, Hao Y, Cai G, Han YW (2013). Fusobacterium nucleatum promotes colorectal carcinogenesis by modulating E-cadherin/β-catenin signaling via its FadA adhesion. Cell Host Microbe.

[63] Wassenaar TME (2018). coli and colorectal cancer: a complex relationship that deserves a critical mindset. Crit Rev Microbiol.

[64] Wu S, Morin PJ, Maouyo D, Sears CL (2003). Bacteroides fragilis enterotoxin induces c-Myc expression and cellular proliferation. Gastroenterology.

[65] Öğrendik M (2015). Oral bacteria in pancreatic cancer: Mutagenesis of the p53 tumour suppressor gene. Int J Clin Exp Pathol.

[66] Whitmore SE, Lamont RJ (2014). Oral Bacteria and Cancer. PLoS Pathog.

[67] Bartpho TS, Wattanawongdon W, Tongtawee T, Paoin C, Kangwantas K, Dechsukhum C (2020). Precancerous Gastric Lesions with Helicobacter pylori vacA +/ babA 2+/ oipA + Genotype Increase the Risk of Gastric Cancer. Biomed Res Int.

[68] Ohnishi N, Yuasa H, Tanaka S (2008). Transgenic expression of Helicobacter pylori CagA induces gastrointestinal and hematopoietic neoplasms in mouse. Chemtracts.

[69] Kwok T, Zabler D, Urman S, Rohde M, Hartig R, Wessler S (2007). Helicobacter exploits integrin for type IV secretion and kinase activation. Nature.

[70] Foegeding NJ, Caston RR, McClain MS, Ohi MD, Cover TL (2016). An overview of Helicobacter pylori VacA toxin biology. Toxins (Basel).

[71] Tombola F, Morbiato L, Del Giudice G, Rappuoli R, Zoratti M, Papini E (2001). The Helicobacter pylori VacA toxin is a urea permease that promotes urea diffusion across epithelia. J Clin Invest.

[72] Oldani A, Cormont M, Hofman V, Chiozzi V, Oregioni O, Canonici A (2009). Helicobacter pylori counteracts the apoptotic action of its VacA toxin by injecting the CagA protein into gastric epithelial cells. PLoS Pathog.

[73] Akada JK, Aoki H, Torigoe Y, Kitagawa T, Kurazono H, Hoshida H (2010). Helicobacter pylori CagA inhibits endocytosis of cytotoxin VacA in host cells. DMM Dis Model Mech.

[74] Yamaoka Y, Kwon DH, Graham DY (2000). A Mr 34,000 proinflammatory outer membrane protein (oipA) of Helicobacter pylori. Proc Natl Acad Sci U S A.

[75] Epplein M, Pawlita M, Michel A, Peek RM, Cai Q, Blot WJ (2013). Helicobacter pylori protein-specific antibodies and risk of colorectal cancer. Cancer Epidemiol Biomarkers Prev.

[76] Butt J, Jenab M, Pawlita M, Tjonneland A, Kyro C, Boutron-Rualt M-C (2020). Antibody Responses to Helicobacter pylori and Risk of Developing Colorectal Cancer in a European Cohort. Cancer Epidemiol Biomarkers Prev.

[77] Bray F, Ferlay J, Soerjomataram I, Siegel RL, Torre LA, Jemal A (2018). Global cancer statistics 2018: GLOBOCAN estimates of incidence and mortality worldwide for 36 cancers in 185 countries. CA Cancer J Clin.

[78] Yan X, Liu L, Li H, Qin H, Sun Z (2017). Clinical significance of Fusobacterium nucleatum, epithelial–mesenchymal transition, and cancer stem cell markers in stage III /IV colorectal cancer patients. Onco Targets Ther.

[79] Burns MB, Lynch J, Starr TK, Knights D, Blekhman R (2015). Virulence genes are a signature of the microbiome in the colorectal tumor microenvironment. Genome Med.

[80] Ganesan K, Guo S, Fayyaz S, Zhang G, Xu B (2019). Targeting programmed fusobacterium nucleatum fap2 for colorectal cancer therapy. Cancers (Basel).

[81] Han YW, Ikegami A, Rajanna C, Kawsar HI, Zhou Y, Li M (2005). Identification and characterization of a novel adhesin unique to oral fusobacteria. J Bacteriol.

[82] Coppenhagen-Glazer S, Sol A, Abed J, Naor R, Zhang X, Han YW (2015). Fap2 of Fusobacterium nucleatum is a galactose-inhibitable adhesin involved in coaggregation, cell adhesion, and preterm birth. Infect Immun.

[83] Kaplan CW, Ma X, Paranjpe A, Jewett A, Lux R, Kinder-Haake S (2010). Fusobacterium nucleatum outer membrane proteins Fap2 and RadD induce cell death in human lymphocytes. Infect Immun.

[84] Tomkovich S, Yang Y, Winglee K, Gauthier J, Mühlbauer M, Sun X (2017). Locoregional effects of microbiota in a preclinical model of colon carcinogenesis. Cancer Res.

[85] Conlin A, Smith G, Carey FA, Wolf CR, Steele RJC (2005). The prognostic significance of K-ras, p53, and APC mutations in colorectal carcinoma. Gut.

[86] Kostic AD, Chun E, Robertson L, Glickman JN, Gallini CA, Michaud M (2014). Fusobacterium nucleatum potentiates intestinal tumorigenesis and modulates the tumor immune microenvironment. Cell Host Microbe.

[87] Purcell R V, Visnovska M, Biggs PJ, Schmeier S, Frizelle FA (2017). Distinct gut microbiome patterns associate with consensus molecular subtypes of colorectal cancer. Sci Rep.

[88] Yeoh YK, Chen Z, Wong MCS, Hui M, Yu J, Ng SC (2020). Southern Chinese populations harbour non-nucleatum Fusobacteria possessing homologues of the colorectal cancer-associated FadA virulence factor. Gut.

[89] Cuevas-Ramos G, Petit CR, Marcq I, Boury M, Oswald E, Nougayrède JP (2010). Escherichia coli induces DNA damage in vivo and triggers genomic instability in mammalian cells. Proc Natl Acad Sci U S A.

[90] Dejea CM, Fathi P, Craig JM, Boleiji A, Taddese R, Geis AL (2018). Patients with familial adenomatous polyposis harbor colonic biofilms containing tumorigenic bacteria. Science.

[91] Raisch J, Buc E, Bonnet M, Sauvanet P, Vazeille E, de Vallée A (2014). Colon cancer-associated B2 Escherichia coli colonize gut mucosa and promote cell proliferation. World J Gastroenterol.

[92] Sedighi M, Zahedi Bialvaei A, Hamblin MR, Ohadi E, Asadi A, Halajzadeh M (2019). Therapeutic bacteria to combat cancer; current advances, challenges, and opportunities. Cancer Med.

[93] Malorni W, Fiorentini C (2006). Is the Rac GTPase-activating toxin CNF1 a smart hijacker of host cell fate?. FASEB J.

[94] Wei Z, Cao S, Liu S, Yao Z, Sun T, Li Y (2016). Could gut microbiota serve as prognostic biomarker associated with colorectal cancer patients’ survival? A pilot study on relevant mechanism. Oncotarget.

[95] Franco AA, Cheng RK, Chung GT, Wu S, Oh HB, Sears CL (1999). Molecular evolution of the pathogenicity island of enterotoxigenic Bacteroides fragilis strains. J Bacteriol.

[96] Sears CL, Geis AL, Housseau F (2014). Bacteroides fragilis subverts mucosal biology: From symbiont to colon carcinogenesis. J Clin Invest.

[97] Rawla P, Sunkara T, Gaduputi V (2019). Epidemiology of Pancreatic Cancer: Global Trends, Etiology and Risk Factors. World J Oncol.

[98] Olsen I, Yilmaz Ö (2019). Possible role of Porphyromonas gingivalis in orodigestive cancers. J Oral Microbiol.

[99] Hayashi C, Gudino CV, Gibson FC, Genco CA (2010). Pathogen-induced inflammation at sites distant from oral infection: bacterial persistence and induction of cell-specific innate immune inflammatory pathways. Mol Oral Microbiol.

[100] Fine DH, Markowitz K, Fairlie K, Tischio-Bereski D, Ferrendiz J, Furgang D (2013). A consortium of Aggregatibacter actinomycetemcomitans, Streptococcus parasanguinis, and Filifactor alocis is present in sites prior to bone loss in a longitudinal study of localized aggressive periodontitis. J Clin Microbiol.

[101] Michaud DS, Joshipura K, Giovannucci E, Fuchs CS (2007). A prospective study of periodontal disease and pancreatic cancer in US male health professionals. J Natl Cancer Inst.

[102] Michaud DS, Izard T (2014). Microbiota, Oral Microbiome and Pancreatic Cancer. Cancer J.

[103] Ahn J, Segers S, Hayes RB (2012). Periodontal disease, Porphyromonas gingivalis serum antibody levels and orodigestive cancer mortality. Carcinogenesis.

[104] Goulas T, Mizgalska D, Garcia-Ferrer I, Kantyka T, Guevara T, Szmigielski B (2015). Structure and mechanism of a bacterial host-protein citrullinating virulence factor. Porphyromonas gingivalis peptidylarginine deiminase Sci Rep.

[105] Gabarrini G, De Smit M, Westra J, Brouwer E, Vissink A, Zhou K (2015). The peptidylarginine deiminase gene is a conserved feature of Porphyromonas gingivalis. Sci Rep.

[106] Gabarrini G, Grasso S, van Winkelhoff AJ, van Dijl JM (2020). Gingimaps: Protein Localization in the Oral Pathogen Porphyromonas gingivalis. Microbiol Mol Biol Rev.

[107] Baugh EH, Ke H, Levine AJ, Bonneau RA, Chan CS (2018). Why are there hotspot mutations in the TP53 gene in human cancers?. Cell Death Differ.

[108] Barton CM, Staddon SL, Hughes CM, O’Sullivan C, Lemoine NR, Hall PA (1991). Abnormalities of the p53 tumour suppressor gene in human pancreatic cancer. Br J Cancer.

[109] Liu L, Wang K, Zhu ZM, Shao JH (2011). Associations between P53 Arg72Pro and development of digestive tract cancers: A meta-analysis. Arch Med Res.

[110] Wu CYC, Carpenter ES, Takeuchi KK, Halbrook CJ, Peverley LV, Bien H (2014). PI3K regulation of RAC1 is required for KRAS-induced pancreatic tumorigenesis in mice. Gastroenterology.

[111] Jiang SN, Phan TX, Nam TK, Nguyen VH, Kim HS, Bom HS (2010). Inhibition of tumor growth and metastasis by a combination of escherichia coli-mediated cytolytic therapy and radiotherapy. Mol Ther.

[112] Bachran C, Hasikova R, Leysath CE, Sastalla I, Zhang Y, Fattah RJ (2014). Cytolethal distending toxin B as a cell-killing component of tumor-targeted anthrax toxin fusion proteins. Cell Death Dis.

[113] Hall EH, Gurel V, Dahlberg AE, McMichael J, Brautigan DL (2011). Inhibition of human breast cancer Matrigel invasion by Streptolysin O activation of the EGF receptor ErbB1. Cell Signal.

[114] Liang ZY, Kang X, Chen H, Wang M, Guan WX (2017). Effect of Clostridium perfringens enterotoxin on gastric cancer cells SGC7901 which highly expressed claudin-4 protein. World J Gastrointest Oncol.

[115] Belibasakis GN, Maula T, Bao K, Lindholm M, Bostanci N, Oscarsson J (2019). Virulence and pathogenicity properties of Aggregatibacter actinomycetemcomitans. Pathogens.

[116] Peng X, Zhang R, Duan G, Wang C, Sun N, Zhang L (2018). Production and delivery of Helicobacter pylori NapA in Lactococcus lactis and its protective efficacy and immune modulatory activity. Sci Rep.

[117] Michalska M, Wolf P (2015). Pseudomonas Exotoxin A: Optimized by evolution for effective killing. Front Microbiol.

[118] Kawakami K, Kawakami M, Husain SR, Puri RK (2002). Targeting interleukin-4 receptors for effective pancreatic cancer therapy. Cancer Res.

[119] Wolf P, Gierschner D, Bühler P, Wetterauer U, Elsässer-Beile U (2006). A recombinant PSMA-specific single-chain immunotoxin has potent and selective toxicity against prostate cancer cells. Cancer Immunol Immunother.

[120] Kumar S, Metz DC, Kaplan DE, Goldberg DS (2020). The association of Helicobacter Pylori with Pancreatic Cancer. GastroHep.

[121] Hirabayashi M, Inoue M, Sawada N, Saito E, Abe SK, Hidaka A (2019). Helicobacter pylori infection, atrophic gastritis, and risk of pancreatic cancer: A population-based cohort study in a large Japanese population: the JPHC Study. Sci Rep.

[122] Nilsson HO, Wadström T, Stenram U, Ihse I (2006). Helicobacter species ribosomal DNA in the pancreas, stomach and duodenum of pancreatic cancer patients. World J Gastroenterol.

[123] Xiao M, Wang Y, Gao Y (2013). Association between Helicobacter pylori Infection and Pancreatic Cancer Development: A Meta-Analysis. PLoS ONE.

[124] Lee WC, Kopetz S, Wistuba II, Zhang J (2017). Metastasis of cancer: When and how?. Ann Oncol.

[125] Parhi L, Alon-Maimon T, Sol A, Nejman D, Shhadeh A, Fainsod-Levi T (2020). Breast cancer colonization by Fusobacterium nucleatum accelerates tumor growth and metastatic progression. Nat Commun.

[126] Kitadai Y (2010). Cancer-Stromal cell interaction and tumor angiogenesis in gastric cancer. Cancer Microenviron.

[127] Idrissi Janati A, Karp I, Sabri H, Emami E (2019). Is a fusobacterium nucleatum infection in the colon a risk factor for colorectal cancer?: A systematic review and meta-analysis protocol. Syst Rev.

[128] Xavier JB, Young VB, Skufca J, Ginty F, Testerman T, Pearson AT (2020). The Cancer Microbiome: Distinguishing Direct and Indirect Effects Requires a Systemic View. Trends in Cancer.

[129] Chen Y, Chen Y, Zhang J, Cao P, Su W, Deng Y (2020). Fusobacterium nucleatum promotes metastasis in colorectal cancer by activating autophagy signaling via the upregulation of CARD3 expression. Theranostics.

[130] Guo S, Chen J, Chen F, Zeng Q, Liu WL, Zhang G (2021). Exosomes derived from Fusobacterium nucleatum -infected colorectal cancer cells facilitate tumour metastasis by selectively carrying miR-1246/92b-3p/27a-3p and CXCL16. Gut.

[131] Salvucci M, Crawford N, Stott K, Bullman S, Longley DB, Prehn JH (2021). Patients with mesenchymal tumours and high Fusobacteriales prevalence have worse prognosis in colorectal cancer (CRC).

[132] Stachowiak R, Lyzniak M, Budziszewska BK, Roeske K, Bielecki J, Hoser G (2012). Cytotoxicity of bacterial metabolic products, including listeriolysin O, on leukocyte targets. J Biomed Biotechnol.

[133] He Z, Gharaibeh RZ, Newsome RC, Pope JL, Dougherty MW, Tomkovich S (2019). Campylobacter jejuni promotes colorectal tumorigenesis through the action of cytolethal distending toxin. Gut.

[134] Wai SN, Lindmark B, Söderblom T, Takade A, Westermark M, Oscarsson J (2003). Vesicle-mediated export and assembly of pore-forming oligomers of the enterobacterial ClyA cytotoxin. Cell.

[135] Sawant SS, Patil SM, Gupta V , Kunda NK, Sciences H (2020). Microbes as Medicines : Harnessing the power of bacteria in advancing cancer treatment. Int J Mo Sci.

[136] Bezine E, Vignard J, Mirey G (2014). The Cytolethal Distending Toxin Effects on Mammalian Cells: A DNA Damage Perspective. Cells.

[137] Haghjoo E, Galán JE (2004). Salmonella typhi encodes a functional cytolethal distending toxin that is delivered into host cells by a bacterial-internalization pathway. Proc Natl Acad Sci U S A.

[138] Kimoto H, Fujii Y, Yokota Y, Taketo A (2005). Molecular characterization of NADase-streptolysin O operon of hemolytic streptococci. Biochim Biophys Acta - Gene Struct Expr.

[139] Kittichotirat W, Bumgarner RE, Asikainen S, Chen C (2011). Identification of the pangenome and its components in 14 distinct aggregatibacter actinomycetemcomitans strains by comparative genomic analysis. PLoS One.

[140] Hill C, Guarner F, Reid G, Gibson GR, Merenstein DJ, Pot B (2014). Expert consensus document: The international scientific association for probiotics and prebiotics consensus statement on the scope and appropriate use of the term probiotic. Nat Rev Gastroenterol Hepatol.

[141] Parisa A, Roya G, Mahdi R, Shabnam R, Maryam E, Malihe T (2020). Anti-cancer effects of Bifidobacterium species in colon cancer cells and a mouse model of carcinogenesis. PLoS One.

[142] Wang Y, Jiang Y, Deng Y, Yi C, Wang Y, Ding M (2020). Probiotic Supplements: Hope or Hype?. Front Microbiol.

[143] Chuah LO, Foo HL, Loh TC, Mohammed Alitheen NB, Yeap SK, Abdul Mutalib NE (2019). Postbiotic metabolites produced by Lactobacillus plantarum strains exert selective cytotoxicity effects on cancer cells. BMC Complement Altern Med.

[144] Park J, Kwon M, Lee J, Park S, Seo J, Roh S. Anti-Cancer Effects of Lactobacillus plantarum L-14 Cell-Free Extract on Human Melanoma A375 Cells. Molecules. 2020;25(17):3895:1–14. 10.3390/molecules25173895. 10.3390/molecules25173895 PMC7503592 32859054

[145] Faghfoori Z, Faghfoori MH, Saber A, Izadi A, Yari KA (2021). Anticancer effects of bifidobacteria on colon cancer cell lines. Cancer Cell Int.

[146] Wei H, Chen L, Lian G, Yang J, Li F, Zou Y (2018). Antitumor mechanisms of bifidobacteria (Review). Oncol Lett.

[147] Yang X, Da M, Zhang W, Qi Q, Zhang C, Han S (2018). Role of Lactobacillus in cervical cancer. Cancer Manag Res.

[148] Le Noci V, Guglielmetti S, Arioli S, Camisaschi C, Bianchi F, Sommariva M (2018). Modulation of Pulmonary Microbiota by Antibiotic or Probiotic Aerosol Therapy: A Strategy to Promote Immunosurveillance against Lung Metastases. Cell Rep.

[149] Nair N, Kasai T, Seno M (2014). Bacteria: Prospective savior in battle against cancer. Anticancer Res.

[150] Brouchkov A, Wang Y, Guo W, Wu XL, Zhang Y, Mannion C (2019). Oncolytic bacteria and their potential role in bacterium-mediated tumour therapy: A conceptual analysis. J Cancer.

[151] Whiteside TL (2008). The tumor microenvironment and its role in promoting tumor growth. Oncogene.

[152] Loeffler M, Le’Negrate G, Krajewska M, Reed JC (2007). Attenuated Salmonella engineered to produce human cytokine LIGHT inhibit tumor growth. Proc Natl Acad Sci U S A.

[153] Kim SH, Castro F, Paterson Y, Gravekamp C (2015). High efficacy of a Listeria-based vaccine against metastatic breast cancer reveals a dual mode of action. Cancer Res.

[154] Roberts NJ, Zhang L, Janku F, Collins A, Bain R-J, Staedtke V (2014). Intratumoral injection of Clostridium noyvi-NT spores induces antitumor responses. SciTranslMed.

[155] Samadi M, Majidzadeh-a K, Salehi M, Jalili N, Noorinejad Z, Mosayebzadeh M (2021). Engineered hypoxia-responding Escherichia coli carrying cardiac peptide genes , suppresses tumor growth , angiogenesis and metastasis in vivo. J Biol Eng.

[156] Duong MTQ, Qin Y, You SH, Min JJ (2019). Bacteria-cancer interactions: bacteria-based cancer therapy. Exp Mol Med.

[157] Pawelek JM, Low KB, Bermudes D (1997). Tumor-targeted Salmonella as a novel anticancer vector. Cancer Res.

[158] Clairmont C, Lee KC, Pike J, Ittensohn M, Low KB, Pawelek J (2000). Biodistribution and genetic stability of the novel antitumor agent VNP20009, a genetically modified strain of Salmonella typhimurium. J Infect Dis.

[159] Toso JF, Gill VJ, Hwu P, Marincola FM, Restifo NP, Schwartzentruber DJ (2002). Phase I study of the intravenous administration of attenuated Salmonella typhimurium to patients with metastatic melanoma. J Clin Oncol.

[160] Li M, Lu M, Lai Y, Zhang X, Li Y, Mao P (2020). Inhibition of acute leukemia with attenuated Salmonella typhimurium strain VNP20009. Biomed Pharmacother.

[161] Zhang Y, Zhang N, Zhao M, Hoffman RM (2015). Comparison of the selective targeting efficacy of Salmonella typhimurium A1-R and VNP20009 on the lewis lung carcinoma in nude mice. Oncotarget.

[162] Hiroshima Y, Zhao M, Zhang Y, Maawy A, Hassanein MK, Uehara F (2013). Comparison of efficacy of Salmonella typhimurium A1-R and chemotherapy on stem-like and non-stem human pancreatic cancer cells. Cell Cycle.

[163] Hiroshima Y, Zhao M, Zhang Y, Zhang N, Maawy A, Murakami T (2015). Tumor-targeting salmonella typhimurium A1-R arrests a chemo-resistant patient soft-tissue sarcoma in nude mice. PLoS ONE.

[164] Kawaguchi K, Igarashi K, Murakami T, Kiyuna T, Zhao M, Zhang Y (2017). Salmonella typhimurium A1-R targeting of a chemotherapy-resistant BRAF-V600E melanoma in a patient-derived orthotopic xenograft (PDOX) model is enhanced in combination with either vemurafenib or temozolomide. Cell Cycle.

[165] Gao S, Jung JH, Lin SM, Jang AY, Zhi Y, Bum Ahn K (2020). Development of Oxytolerant Salmonella typhimurium Using Radiation Mutation Technology (RMT) for Cancer Therapy. Sci Rep.

[166] Janku F, Zhang HH, Pezeshki A, Goel S, Murthy R, Wang- Gillam A (2021). Intratumoral injection of clostridium novyi-NT spores in patients with treatment-refractory advanced solid tumors. Clin Cancer Res.

[167] Mazor R, Pastan I (2020). Immunogenicity of Immunotoxins Containing Pseudomonas Exotoxin A: Causes Consequences and Mitigation. Front Immunol.

[168] Dieffenbach M, Pastan I (2020). Mechanisms of resistance to immunotoxins containing pseudomonas exotoxin a in cancer therapy. Biomolecules.

[169] Alewine C, Ahmad M, Peer CJ, Hu ZI, Lee M-J, Yuno A (2020). Phase I/II Study of the Mesothelin-targeted Immunotoxin LMB-100 with Nab-Paclitaxel for Patients with Advanced Pancreatic Adenocarcinoma. Clin Cancer Res.

[170] Weerakkody LR, Witharana C (2019). The role of bacterial toxins and spores in cancer therapy. Life Sci.

[171] Scott AJ, Alexander JL, Merrifield CA, Cunningham D, Jobin C, Brown R (2019). International Cancer Microbiome Consortium consensus statement on the role of the human microbiome in carcinogenesis. Gut.

